# Er:YAG Laser Applications for Debonding Different Ceramic Restorations: An In Vitro Study

**DOI:** 10.3390/medicina61071189

**Published:** 2025-06-30

**Authors:** Ruxandra Elena Luca, Anișoara Giumancă-Borozan, Iosif Hulka, Ioana-Roxana Munteanu, Carmen Darinca Todea, Mariana Ioana Miron

**Affiliations:** 1 University Clinic of Oral Rehabilitation and Dental Emergencies, Faculty of Dentistry, “Victor Babes” University of Medicine and Pharmacy, Eftimie Murgu Square No. 2, 300041 Timisoara, Romania; luca.ruxandra@umft.ro (R.E.L.); todea.darinca@umft.ro (C.D.T.); miron.mariana@umft.ro (M.I.M.); 2 Interdisciplinary Research Center for Dental Medical Research, Lasers and Innovative Technologies, Revolutiei 1989 Avenue No. 9, 300070 Timisoara, Romania; 3 Faculty of Dentistry, “Victor Babes” University of Medicine and Pharmacy Graduate, 300041 Timisoara, Romania; aniy_borozan@yahoo.com; 4 Research Institute for Renewable Energies, University Politehnica Timișoara, G. Muzicescu 138, 300501 Timișoara, Romania; iosif.hulka@upt.ro

**Keywords:** ceramics, debonding, electron microscopy, Er:YAG laser

## Abstract

*Background and Objectives*: Conventional methods for removing cemented fixed prosthetic restorations (FPRs) are unreliable and lead to unsatisfactory outcomes. At their best, they allow the tooth to be saved at the expense of a laborious process that also wears down rotating tools and handpieces and occasionally results in abutment fractures. Restorations are nearly never reusable in any of these situations. Erbium-doped yttrium-aluminum-garnet (Er:YAG) and erbium-chromium yttrium-scandium-gallium-garnet (Er,Cr:YSGG) lasers casafely and effectively remove FPRs, according to scientific studiesre. This study sets out to examine the impact of Er:YAG laser radiation on the debonding of different ceramic restorations, comparing the behavior of various ceramic prosthetic restoration types under laser radiation action and evaluating the integrity of prosthetic restorations and dental surfaces exposed to laser radiation. *Materials and Methods*: The study included a total of 16 removed teeth, each prepared on opposite surfaces as abutments.y. Based on the previously defined groups, four types of ceramic restorations were included in the study: feldspathic (F), lithium disilicates (LD), layered zirconia (LZ), and monolithic zirconia (MZ). The thickness of the prosthetic restorations was measured at three points, and two different materials were used for cementation. The Er:YAG Fotona StarWalker MaQX laser was used to debond the ceramic FPR at a distance of 10 mm using an R14 sapphire tip with 275 mJ, 20 Hz, 5.5 W, with air cooling (setting 1 of 9) and water. After debonding, the debonded surface was visualized under electron microscopy. *Results*: A total of 23 ceramic FPRs were debonded, of which 12 were intact and the others fractured into two or three pieces. The electron microscopy images showed that debonding took place without causing any harm to the tooth structure. The various restoration types had the following success rates: 100% for the LZ and F groups, 87% for the LD group, and 0% for the MZ group. In terms of cement type, debonding ceramic FPRs cemented with RELYX was successful 75% of the time, compared to Variolink DC’s 69% success rate. *Conclusions*: In summary, the majority of ceramic prosthetic restorations can be successfully and conservatively debonded with Er:YAG radiation.

## 1. Introduction

Traditional techniques for removing permanently cemented fixed prosthetic restorations (FPRs) do not provide reliable and satisfactory results. At their best, they allow tooth preservation at the cost of a time-consuming procedure, causing wear of rotary instruments and handpieces or even abutment fractures. The restorations are seldom reusable, which is a disadvantage for both patient and physician in the event of incorrect cementation [1].

The obstacles encountered are common to all conventional removal of prosthetic restorations procedures, and they include: the retention capacity, which is conditioned by the type and shape of the preparation, the contact surface between the abutment and the prosthetic device, the cement type, and the form and structure of the abutment. Regardless of the method used to remove FPRs, the dentist faces several significant challenges, including the following: some of the pressure exerted by the instrument is absorbed by the periodontal ligament, with the risk of pain and even luxation; in cases of massive reconstruction, there is an increased risk of fracturing the reconstruction [1].

There is currently no ideal conventional removal system [1,2,3,4,5], and its choice depends on the type of FPR, the cement used, the type of preparation, the abutment (natural or reconstructed tooth), and its position in the oral cavity [2].

Depending on the method of operation, techniques for removing prosthetic works can be classified as follows [2]:

- very conservative: ultrasonic, FPR Removal Thermoplastic Adhesive Resin, lasers, and FPR forceps;
- conservative: various manual, semi-automatic, automatic, or pneumatic removal instruments;
- semi-conservative: Metalift, Kline, WAMkey, or Higa systems;
- invasive: in which FPRs are sectioned with diamond or tungsten carbide burs [2,3], after which different forceps or removal instruments are applied.

No universal system can ensure intact and safe removal of classically cemented FPRs; therefore, combining several systems seems to be a better option, but with very little chance of reusing the prosthetic piece.

Since the introduction of the laser into prosthetic dentistry, a new era has opened, with an impact on the quality of treatment and the hope of overcoming the disadvantages of conventional methods. Laser technology has many advantages: it causes less or no pain; eliminates the noise of the traditional instruments that represents a source of anxiety for some patients; is minimally invasive and precise, offering rapid healing without vibrations; helps achieve immediate hemostasis and moisture control; and is being successfully used in patients with phobias or allergies to local anesthetics [6]. Lasers have been introduced in every area of dentistry: oral surgery, general dentistry, pedodontics, endodontics, periodontology, implantology, and prosthetics [6]. In fixed restorations, removing FPRs without damaging tooth structure is difficult and time-consuming [2,4,7,8]. Due to the disadvantages of conventional methods, other methods have been sought to remove FPRs safely, predictably, and quickly, Er:YAG and Er,Cr:YSGG being among them [2,8,9].

According to numerous studies, the effectiveness of the Er:YAG laser in debonding FPRs is achieved without damaging the tooth structure, measuring the energy used and the time required [6,8,9,10,11], but the parameters are contradictory, and the results are uneven in various studies.

The Er laser family exists between 2780 nm (Er,Cr:YSGG) and 2940 nm (Er:YAG), and these wavelengths are well absorbed in water and hydroxyapatite. This type of laser wavelength has been used experimentally since the early 1990s to remove the ceramic orthodontic brackets [12]. As Er:YAG lasers demonstrated their effectiveness in the debonding of brackets, the studies were continued in the case of ceramic FPR debonding.

The detachment of ceramic FPRs is achieved by the H₂O/O₂ absorption band, which coincides with the wavelength of the Er:YAG laser. Not all ceramic FPRs can absorb the wavelength; therefore, the laser energy is transmitted through the FPR, displacing the bonding cement. Most FPRs can be removed intact, which is useful in the event of a cementation error. Flexural strength is key to maintaining the integrity of ceramic FPRs during laser debonding [10,11,13].

Laser debonding of ceramic FPRs has many advantages but can be affected by a variety of clinical factors, including the chemical composition and type of ceramic, restoration thickness, type and shade of resin cement, ceramic shade and opacity, material porosity, and laser parameters (power, pulse, irradiation time) [8,12]. Probably due to the high variability of all these factors that can influence the outcome, studies report different results. For example, according to Morford’s studies [10], it has been demonstrated that FPRs can be debonded using the Er:YAG (2940 nm) and Er,Cr:YSGG (2780 nm) lasers, provided the FPRs are made of ceramic masses, but these lasers are not effective in the case of FPRs made of zirconia or with a metal component. On the other hand, the studies of Rechmann et al. [11] concluded that Er:YAG can be effective in removing both ceramic and zirconium oxide FPRs. The use of laser energy in FPR debonding is based on the absorption of energy by the cement, causing its degradation through three mechanisms: thermal degradation, thermal ablation, and photoablation [14]. The laser energy is transferred through the veneers and crowns, which then react with the cement, damaging it, followed by the detachment of the FPR from the tooth [11].

The device parameters and the correct laser action technique applied to the cement–ceramic interface are essential in trying not to cause additional damage to the tooth structure. The parameters of the Er:YAG laser undergo changes dictated by the type of ceramic FPR and its thickness, as well as the properties of the adhesive material [15].

The purpose of this study is to address the following areas of interest:

(1) Examine the influence of the Er:YAG laser used for the debonding of different types of ceramic FPRs.
(2) Perform a comparative analysis of the behavior of different types of ceramic prosthetic restorations and cements under the action of laser radiation.
(3) Analyze the integrity of prosthetic restorations and tooth surfaces subjected to the action of laser radiation.

## 2. Materials and Methods

**Figure 1:**
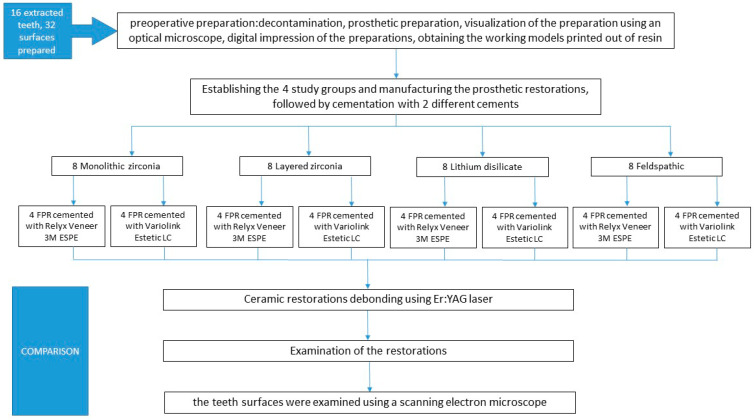
A flowchart showing study groups and working protocol.

The research was conducted at the “Victor Babeș” University of Medicine and Pharmacy Timișoara, in collaboration with the Politehnica University Timisoara, briefly described by the flowchart shown in Figure 1.

**Figure 2:**
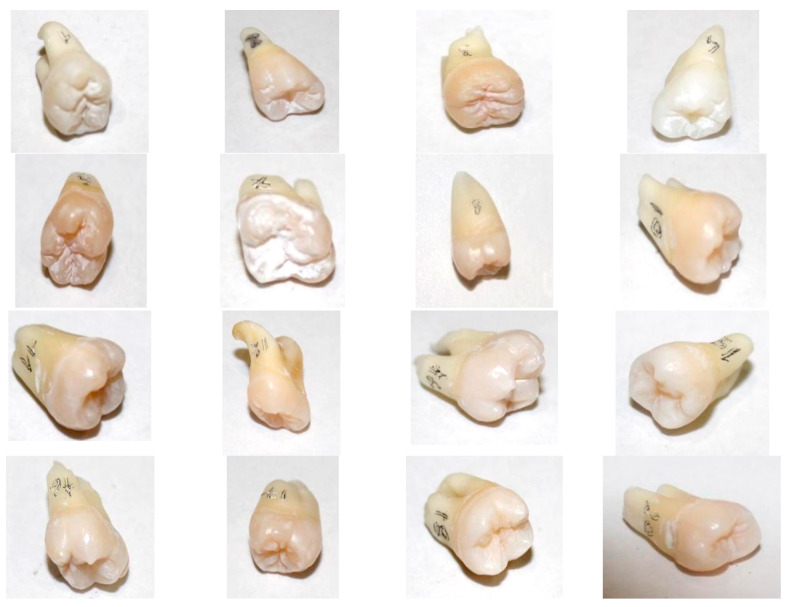
The 16 decontaminated lateral teeth, ready for preparation.

We used 16 extracted teeth (Figure 2) that met the inclusion criteria in the study: no caries lesion on the surfaces to treat and no endodontic or restorative treatment performed previously.

**Figure 3:**
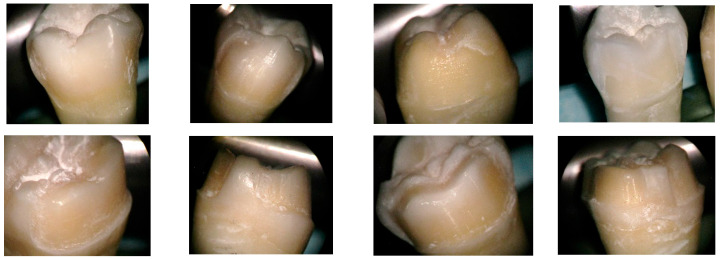
Prepared tooth surface visualized with an optical microscope, magnification 20×.

**Figure 4:**
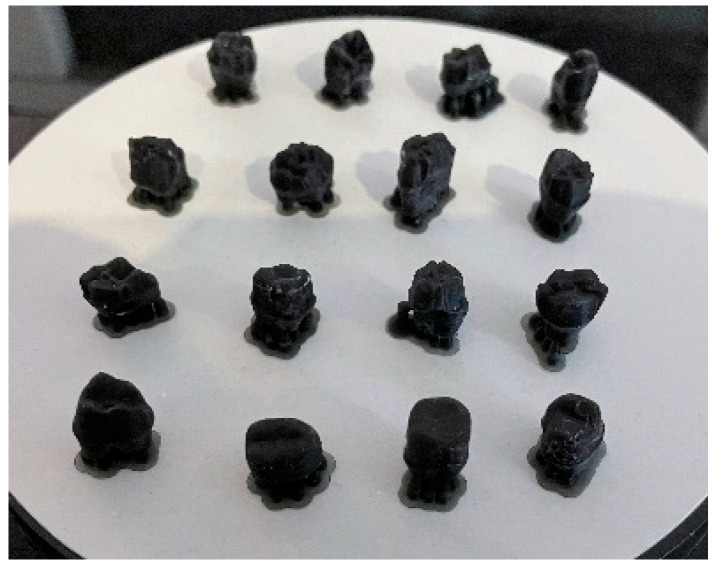
Working models printed with black resin.

**Figure 5:**
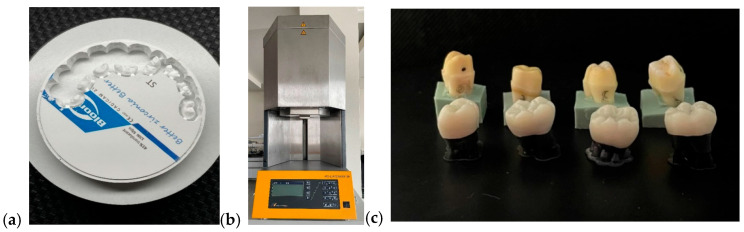
Unsintered milled ceramic FPR (**a**), sintering furnace (**b**), monolithic zirconia restorations (**c**).

**Figure 6:**
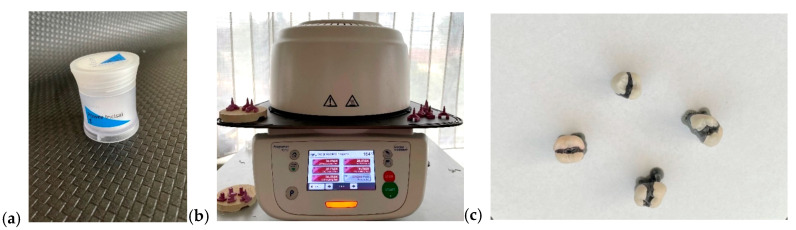
Emax Ceram (**a**), Ivoclar Vivadent Programmed P310 oven (**b**), layered zirconia restorations (**c**).

**Figure 7:**
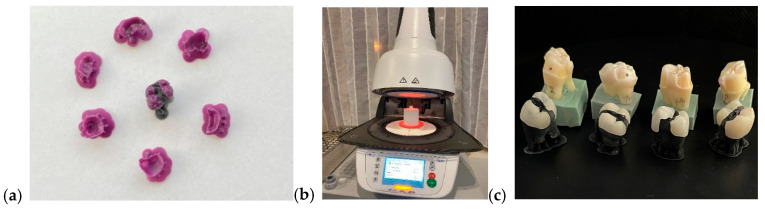
Castable resin models (**a**), Ivoclar Vivadent Programmed P310 oven (**b**), lithium disilicate restorations (**c**).

**Figure 8:**
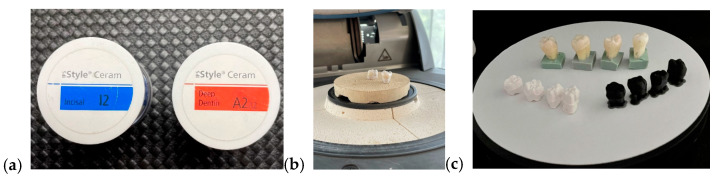
IPS Style Ceram (**a**), feldspathic restorations on refractory abutment (**b**), feldspathic restorations (**c**).

The teeth underwent several stages of preoperative preparation, which are explained below:

⮚ Decontamination
  - immersion in 5.25% sodium hypochlorite for 15 min
  - immersion in saline solution for 24 h
  - immersion in distilled water for 7 days
⮚ Preparation
  - Equipment: diamond cylindrical–conical burs of grain size 20–30 μ and 125–150 μ, and a guide bur of grain size 106–125 μ, with a diameter of 1 mm, using a Kavo high-speed handpiece, under air-water cooling;
  - The preparation thickness was measured at 3 points: incisal, middle, and cervical third.
⮚ Visualization of the preparation was performed using an optical microscope (Zeiss Surgical GmbH, Oberkochen, Germany) at magnifications of 20× and 30× to assess the enamel surface (Figure 3), the photos being taken with a Nikon camera with an AF-S Micro NIKKOR 85 mm 1:3.5 G lens (Nikon, Tokyo, Japan); examination of the prepared surface was performed using a scanning electron microscope (SEM: Quanta FEG 250, FEI, Hillsboro, OR, USA) to assess structural integrity and identify any potential surface damage. The SEM was operated in low-vacuum mode to avoid surface charging, using secondary electrons to highlight the topography of the surface. The micrographs were collected at 10 kV with a working distance of about 10 mm. Representative micrographs of the surface morphological changes were captured at 1000× and 2000× magnification.
⮚ Digital impression of the preparations was achieved using a MEDIT T-300 scanner (Medit, Seoul, Republic of Korea) and Exocad Dental DB 2.2 Valletta software (exocad GmbH, Darmstadt, Germany);
⮚ Working models printed out of resin were obtained with an Anycubic 3D printing UV-sensitive resin–black (Hongkong Anycubic Technology Co., Hong Kong, China) on a Phrozen 4K printer (Phrozen Tech Co., Ltd., Hsinchu City, Taiwan) (Figure 4).
⮚ The fixed prosthetic restorations from four types of ceramics were manufactured as follows:

I. Monolithic zirconia, STML, milled with an Imes-icore250i machine and sintered at a temperature of 1350 °C in a Mihmvogt HTS-2/M/ZIRKON-120 furnace (MIHM-VOGT GmbH & Co. KG, Stutensee-Blankenloch, Germany) (Figure 5).
II. Layered zirconia: FPR framework made of zirconia, layered with Emax Ceram ceramic, sintered at 770 °C in an Ivoclar Vivadent Programat P310 furnace (Ivoclar, Schaan, Liechtenstein) (Figure 6).
III. Lithium disilicate: Emax press packed and pressed at 925 °C in an Ivoclar Vivadent Programat EP 3010 furnace (Ivoclar, Schaan, Liechtenstein) (Figure 7).
IV. Feldspathic FPR on refractory abutment: IPS Style Ceram ceramic, layered on a working model made of refractory mass and sintered at 790 °C in the Ivoclar Vivadent Programat P310 furnace (Figure 8).

**Figure 9:**
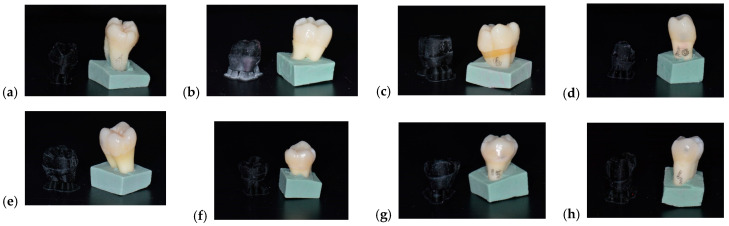
Cemented ceramic prosthetic restorations (**a**–**h**).

⮚ Evaluation of the ceramic restorations’ thickness in 3 points, using a micrometer: incisal third, middle third, and cervical third.
⮚ Cementation of the prosthetic restorations, corresponding to one of the study groups (Figure 9):

A. Relyx U2000 3M ESPE:
  - protocol for the prosthetic restoration: HF acid etching (30–60 s)/sandblasting (depends on material)
  - tooth protocol: no prior preparation required
  - cement application and light curing, 30 s on all surfaces
B. Variolink Estetic DC
  - protocol for the prosthetic restoration: HF acid etching (30–60 s)/sandblasting (depends on material), Monobond (application for 1 min)
  - tooth protocol: application of Single Bond Universal Adhesive 3M (polymerization, 30 s)
  - cement application and light polymerization, 30 s on all surfaces

**Figure 10:**
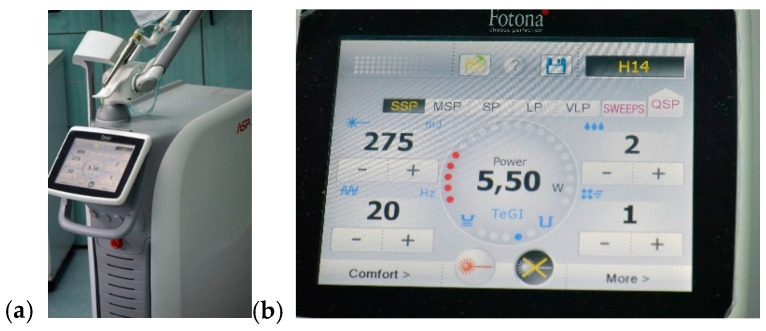
Er:YAG laser (**a**), established parameters for debonding procedure (**b**).

**Figure 11:**
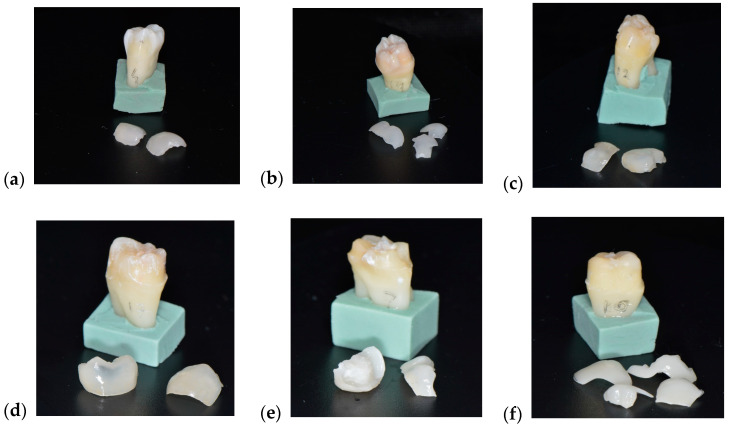
Aspects of teeth and restorations after laser debonding (**a**–**f**).

⮚ After cementation, the ceramic prosthetic restorations were placed in saline for 72 h.
  Ceramic restorations debonding: It was performed using an Er:YAG laser (Fotona StarWalker MaQX laser, Fotona d.o.o. Ljubljana, Slovenia) with a R14 sapphire tip at a distance of 10 mm, set at 275 mJ, 20 Hz, 5.5 W with air cooling (setting 1 of 9) and water (setting 2 of 9); the optimal parameters were determined following testing on ceramic materials cemented on a pilot support (Figure 10 and Figure 11), in accordance with similar data from the literature [8,15].
⮚ Following the debonding process, the teeth surfaces were examined using a scanning electron microscope (SEM: Quanta FEG 250, FEI, Hillsboro, OR, USA) to assess structural integrity and identify any potential surface damage. The SEM was operated in low-vacuum mode to avoid surface charging, using secondary electrons to highlight the topography of the surface. The micrographs were collected at 10 kV with a working distance of about 10 mm. Representative micrographs of the surface morphological changes were captured at 1000× and 2000× magnification.

## 3. Results

### 3.1. Measurement of the Four Different Types of Restorations Thickness

The thickness of the ceramic restorations was assessed at three anatomical landmarks—cervical, middle, and occlusal thirds—across four material types: monolithic zirconia (Table 1), layered zirconia (Table 2), lithium disilicate (Table 3), and feldspathic ceramic (Table 4). For each sample, two fixed partial restorations (FPR 1 and FPR 2) were measured, and average thickness values were calculated.

Monolithic zirconia exhibited the highest thickness values across all measurement points. The mean occlusal thickness reached 2.38 mm, with an average total restoration thickness of 1.39 mm ± 0.30. These values were notably higher than those observed in any other group. Importantly, none of the monolithic zirconia samples were successfully debonded under the applied Er:YAG laser parameters. 

The layered zirconia group presented an average restoration thickness of 0.78 mm ± 0.18, with a mean occlusal thickness of 1.11 mm. Despite the moderate occlusal bulk, all restorations in this group were successfully debonded. 

Lithium disilicate restorations demonstrated an average thickness of 0.79 mm ± 0.23, comparable to layered zirconia. However, individual occlusal measurements reached up to 1.70 mm, which may explain the slightly lower debonding success rate of 87%. 

Feldspathic ceramic was the thinnest among all groups, with a mean total thickness of 0.60 mm ± 0.18 and minimal variation between samples. All feldspathic restorations were successfully debonded. 

### 3.2. Sample Centralizer

The following table (Table 5) presents the situations of all samples included in the study, in terms of the type of prosthetic restoration and measured parameters (thickness, surface of the prosthetic restoration), as well as the results of applied laser radiation using the following process parameters: 275 [mJ], 20 [Hz] and 5.5 W for all the samples.

**Figure 12:**
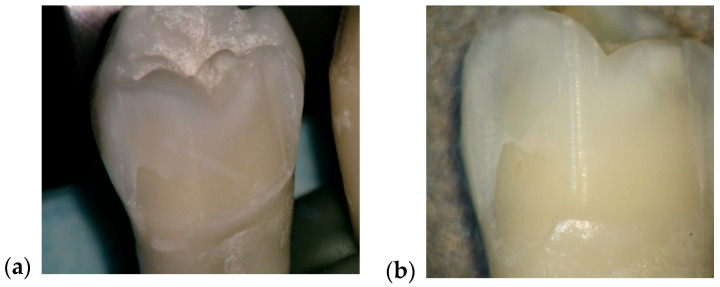
Comparative aspect of enamel surface before and after laser debonding, visualized with the optical microscope, at magnifications of 20× and 30× (**a**,**b**).

**Figure 13:**
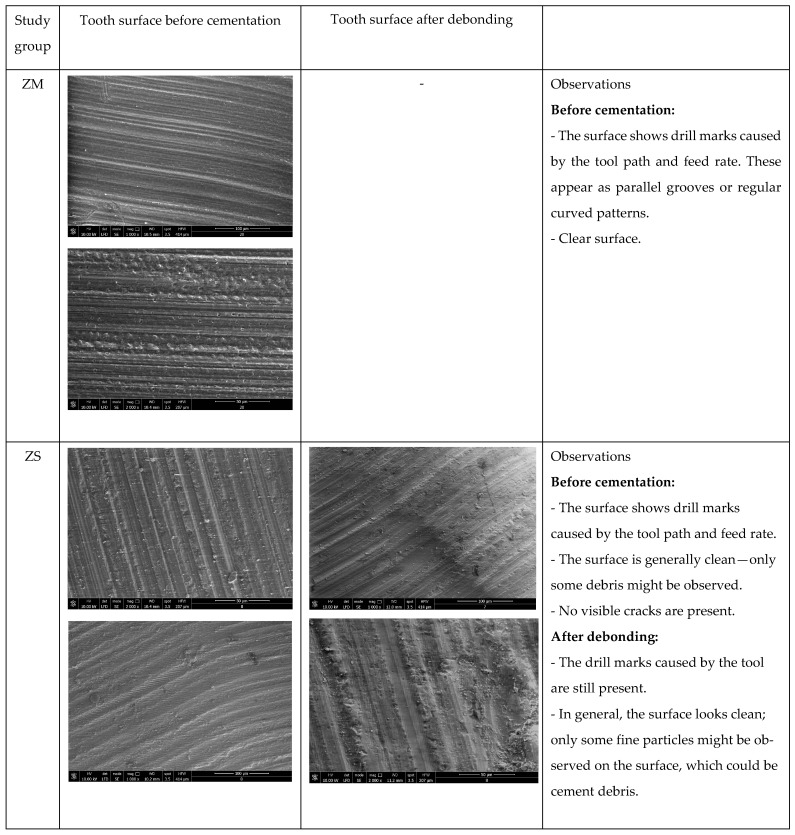

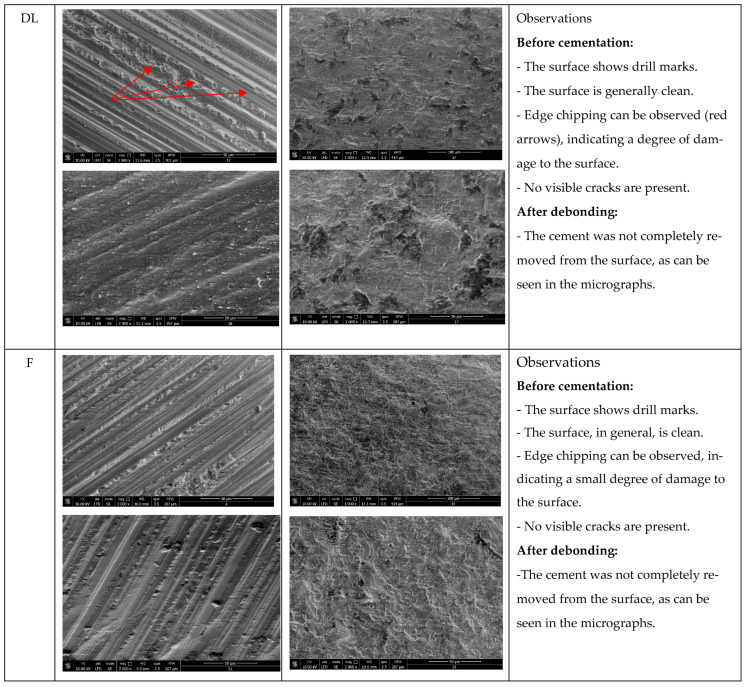
Representative images of the tooth surface under SEM, before cementation and after debonding (organized as per study group depending on the ceramic restoration type), 1000× and 2000× magnification.

Following the experiment, 23 ceramic restorations were debonded, of which 12 were intact and the others fractured into two or three pieces. The debonding occurred without damaging the tooth structure, according to images obtained with the optical microscope, at magnifications of 20× and 30× (Figure 12) and with a scanning electron microscope at 10 kV, with a working distance of about 10 mm. Representative micrographs of the surface morphological changes were captured at 1000× and 2000× magnification (Figure 13).

#### 3.2.1. Debonding Success Rate by Ceramic Type

**Figure 14:**
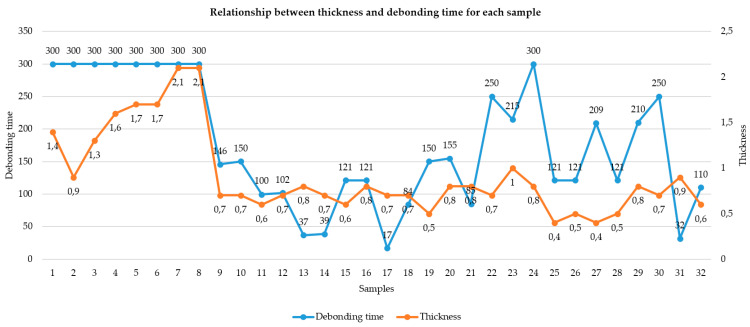
Thickness–debonding chart.

Laser-assisted debonding was successful in 23 out of 32 restorations (71.9%). A clear threshold was identified in relation to restoration thickness: no restoration with a thickness exceeding 1.0 mm was successfully debonded within the 5 min irradiation window. Conversely, restorations with a thickness of 1.0 mm or less consistently responded to laser irradiation (see Figure 14).

#### 3.2.2. Influence of Cement Type on Debonding Success

Among the tested restorations, 16 were luted with Relyx U200 (3M, North Ryde, Australia) and 16 with Variolink Esthetic DC (Ivoclar Vivadent AG, Schaan, Liechtenstein). Debonding was successful in 75% of the Relyx group and 69% of the Variolink group.

A chi-square test for independence revealed no statistically significant association between cement type and debonding outcome (χ^2^ = 0.14, *p* > 0.05). Nonetheless, the slightly higher success rate in the Relyx group suggests that cement composition and polymerization profile may influence thermal degradation behavior under Er:YAG laser exposure.

#### 3.2.3. Effect of Ceramic Thickness on Debonding Outcome

Restoration thickness was found to be a critical determinant in the success of laser-assisted ceramic debonding. Among the 32 restorations included in the study, all samples with a total thickness exceeding 1.0 mm failed to debond, despite the use of standardized laser settings (275 mJ, 20 Hz, 5.5 W for 5 min). Conversely, 100% of the restorations with thicknesses ≤ 1.0 mm were successfully debonded, establishing a clearly defined threshold for clinical success.

This binary outcome underscores the strong and categorical influence of ceramic thickness on the ability of Er:YAG laser energy to penetrate the prosthetic structure and reach the resin cement interface. Statistical analysis and visual inspection (Figure 14) further support the presence of this threshold effect, with no overlap between successful and failed groups in terms of thickness.

Anatomically, this limitation was most pronounced in the occlusal third, where ceramic bulk is generally greatest. Monolithic zirconia restorations, which exhibited the highest occlusal thickness values (mean: 2.38 mm), were uniformly resistant to debonding. In contrast, feldspathic ceramics, which maintained the lowest thickness across all thirds (mean: 0.60 mm), showed consistent and complete debonding success.

To determine whether restoration thickness was significantly different between debonded and non-debonded samples, we performed both an independent samples *t*-test and a Mann–Whitney U test following verification of data distribution.

Using the Shapiro–Wilk test, the distribution of thickness values was found to be approximately normal in both groups:

- Debonded group: W = 0.945, *p* = 0.228
- Not debonded group: W = 0.932, *p* = 0.503

As both *p*-values exceeded 0.05, the assumption of normality was upheld, justifying the use of the *t*-test for group comparison.

##### *t*-Test Results

The independent samples *t*-test revealed a highly significant difference in mean thickness:

- t(df) = −5.30, *p* = 0.0006

This confirms that restorations that failed to debond were significantly thicker than those successfully debonded.

##### Mann–Whitney U Test (Validation)

To confirm this result with a non-parametric method, the Mann–Whitney U test was also conducted:

- U = 6.0, *p* < 0.0001

The non-parametric test strongly supported the *t*-test conclusion, reinforcing the robustness of the result regardless of distribution assumptions.

These tests confirm that thickness is not only clinically but also statistically a significant barrier to successful debonding. The finding that no restoration > 1.0 mm was successfully removed, and that the mean thickness in failed cases was significantly higher, provides strong justification for emphasizing design limitations and clinical thresholds in ceramic restoration planning.

#### 3.2.4. Correlation Between Surface Area and Debonding Time

To evaluate the influence of geometric parameters on the duration of laser-assisted debonding, correlation and linear regression analyses were conducted on the 23 restorations that were successfully debonded.

##### Thickness vs. Debonding Time

The Pearson correlation between restoration thickness and debonding time was found to be negligible (r = −0.067, *t*(21) = −0.31, *p* = 0.76). A linear regression model yielded the following equation:

$$Debonding\;time\;\left(s\right)=-29.56\times Thickness\;\left(mm\right)+148.14$$

The model’s explanatory power was minimal, with a coefficient of determination (R^2^) of 0.0045, indicating that thickness accounted for less than 0.5% of the variation in debonding duration.

##### Surface Area vs. Debonding Time

Similarly, the correlation between restoration surface area and debonding time was weak and non-significant (*r* = −0.045, *t*(21) = −0.21, *p* = 0.84). The corresponding linear regression model was

$$Debonding\;time\;\left(s\right)=-0.31\times surface\;area\;\left({mm}^{2}\right)+142.03$$

The R^2^ value for this model was only 0.0020, confirming an absence of meaningful predictive power.

#### 3.2.5. Restoration Integrity Post-Debonding

Of the 23 restorations that were successfully debonded,

- 12 (52%) remained intact
- 9 (39%) fractured during laser exposure
- 2 (9%) fractured during clinical handling

These proportions were independent of cement or ceramic type and suggest that laser debonding can preserve restoration integrity in approximately half of the cases, offering the possibility of reuse in clinical corrections (e.g., fit adjustment, color correction).

#### 3.2.6. Ceramic Type Analysis

To assess the relationship between ceramic material and debonding success, a chi-square test of independence was performed. The analysis included all 32 restorations, equally distributed across four ceramic categories: monolithic zirconia, layered zirconia, lithium disilicate, and feldspathic ceramic, with 8 restorations in each group (Table 6).

To further investigate the impact of ceramic composition on debonding success, Fisher’s exact tests were conducted across all four material types (Table 7). These comparisons revealed that monolithic zirconia (MZ) performed significantly worse than all other ceramics, with 0% success under standardized laser conditions. In contrast, layered zirconia (LZ) and feldspathic ceramics (F) both demonstrated 100% success, while lithium disilicate (LD) achieved 87.5%.

Statistical testing confirmed that the differences between monolithic zirconia and every other group were highly significant (*p* < 0.001). However, no statistically significant differences were found among LZ, LD, and F (all *p* ≈ 1.0), indicating comparable clinical behavior in terms of retrievability.

## 4. Discussion

According to our study data, the factor that directly influences the debonding process by laser irradiation is the thickness of the ceramic FPR. Ceramic restorations with a thickness of over 1 mm could not be debonded at an irradiation of 5 min, 275 mJ, 20 Hz, and 5.5 W (Figure 14). This binary outcome was confirmed by a significant difference in mean thickness between debonded and non-debonded groups (*p* < 0.001, t = −5.30), establishing 1.0 mm as a critical clinical threshold.

Our results suggest that laser energy is both geometrically and optically dependent, with thicker and more crystalline structures attenuating energy transmission to a degree that renders debonding ineffective.

Furthermore, the finding that restorations near the 1.0 mm cutoff in lithium disilicate and layered zirconia showed occasional delays in debonding supports the hypothesis that even small increases in thickness can significantly reduce clinical efficiency, especially in materials with moderate opacity.

Conversely, restorations with a thickness of 1.0 mm or less consistently responded to laser irradiation, supporting the conclusion that ceramic thickness is a primary determinant of successful laser-assisted debonding under fixed energy parameters.

From a clinical standpoint, this evidence reinforces the need to limit ceramic thickness—particularly in the occlusal third—when planning restorations in situations where future retrievability may be required. Restoration design protocols may benefit from incorporating retrievability consideration during digital planning, especially in the case of adhesive cementation.

**Figure 15:**
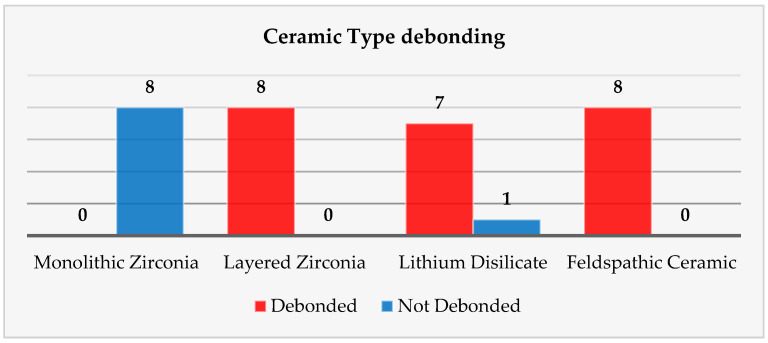
The ceramic’s type influencing the debonding process chart.

When analyzing the thickness in relation to different types of ceramics, several important conclusions can be drawn (Figure 15). When observing the monolithic zirconia group (not debonded), the outcome supports the hypothesis that high material density and excessive thickness significantly attenuate laser energy before it can reach and soften the underlying resin cement. Regarding layered zirconia, all samples were successfully debonded, suggesting that the material’s layered structure and relatively lower opacity allowed for sufficient laser transmission and energy absorption at the cement interface, facilitating effective debonding even at slightly greater thicknesses. The lithium disilicate group showed increased occlusal measurements; these thicker occlusal zones could have limited laser penetration and contributed to resistance during the debonding process.

Feldspathic ceramic samples, the thinnest, were also successfully debonded. This finding is consistent with the high optical transmittance and low structural density of feldspathic ceramics, which allow efficient laser energy propagation to the adhesive interface with minimal attenuation.

Taken together, these data confirm that ceramic thickness is a critical variable in laser-assisted debonding. While composition and translucency play key roles, restorations exceeding 1.0 mm in occlusal thickness consistently showed reduced laser efficiency, particularly in dense materials such as monolithic zirconia. This reinforces the clinical recommendation to consider both material selection and design thickness when planning restorations intended for potential laser retrieval. This finding aligns with previous literature indicating that laser energy is significantly attenuated when passing through thicker and denser ceramics, especially in materials with low translucency, such as monolithic zirconia.

From a clinical perspective, these findings emphasize the importance of material selection in cases where retrievability is a concern. Highly dense and low-translucency materials such as monolithic zirconia may not be suitable for laser-assisted retrieval protocols, while more translucent ceramics demonstrate predictable removal and may support future reuse.

Regarding the choice of power, we previously explained that, in our study, this was achieved after repeated trials, with different values, on ceramic blocks of different thicknesses. The literature does not offer a consensus in this direction, although the settings are comparable. For example, a study to which we can refer [16] comparatively investigated powers of 4, 5, and 6 W used to debond novel zirconia restorations; in fact, the restorations were not cemented, but only laser radiation was applied to them, in order to investigate possible structural changes in the prosthetic restorations. The conclusion of the study was that Er:YAG laser debonding did not affect the optical or mechanical properties of dental zirconia ceramics and did not cause any microcracks of the zirconia ceramic surfaces. Clinical studies have also shown the lack of side effects on abutment teeth, especially when the laser is used with appropriate cooling parameters [10,11,17]. From a clinical point of view, this could be of real help, especially considering the discomfort reported by patients regarding conventional removal techniques. Studies were conducted in this regard, aiming to determine the most effective methods in controlling pain during debonding procedures [18].

**Figure 16:**
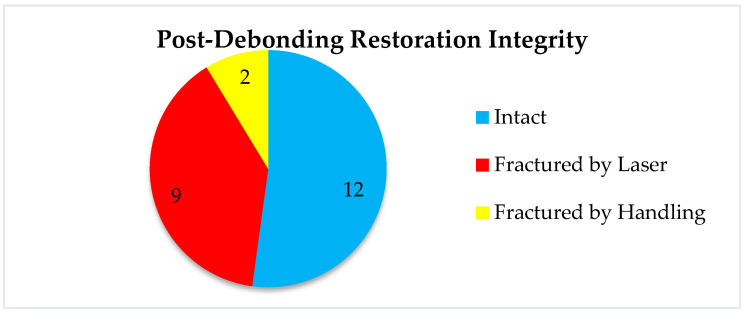
Post-debonding restoration integrity chart.

Another very interesting study measured the rebonding strengths of 1 mm thickness leucite and lithium disilicate veneers after the debonding process using the Er:YAG laser and rebonding, respectively. The conclusions of the study showed statistically significant differences between the control group, in which no laser was used, and the one in which the debonding was performed using the laser. The Er:YAG laser demonstrated the ability to debond restorations with a power of 3 W, 10 Hz, and 300 mJ, with 100 μs pulse duration for 9 s, and rebonding did not influence the shear bond strength [19]. This information is very important in all cases when retrievability as a concern. Our study aimed not only at the laser debonding efficacy but also at the reusability of the prosthetic restorations, i.e., the condition of the prosthetic piece after debonding. Following laser debonding, 52% of prosthetic restorations were intact, (which helps when we consider retrievability), 48% were fractured/cracked, of which 39% were fractured/cracked following irradiation, and 9% were fractured due to improper handling during cementation (Figure 16). El-Sheikh et. al. [20] evaluated three ceramic types of 0.5 mm thickness debonded using Er;Cr:YSGG. In terms of debonded veneer damage, the Emax and zirconia samples revealed none. However, 40% of the LiSi samples fractured during debonding and 20% had cracks. Zhang et. al. [21] investigated how different types of ceramics were affected by Er:YAG laser irradiation, in terms of optical and mechanical properties. They concluded that while mechanical properties remain unchanged, the optical ones are affected by laser radiation of greater power, causing perceptible color changes. The selection of appropriate laser parameters is therefore very important, especially in cases of retrievability. Another aspect investigated in a recent experimental study was that of roughness modifications [22]. The authors concluded that lasers are a noninvasive way to retrieve ceramic veneers, although surface roughness modifications were found in both types of investigated ceramics.

Several studies extend the applicability from the tooth level to the similar situation of the implant-supported prosthetic restorations debonding [23,24,25,26]. Studies revealed that both the Er:YAG and Er,Cr:YSGG lasers work similarly to remove a zirconia crown from a zirconia implant abutment with identical parameters, even though they operate at different wavelengths. The authors concluded that pulsed erbium laser irradiation does not cause any damage to the optical or elemental composition.

**Figure 17:**
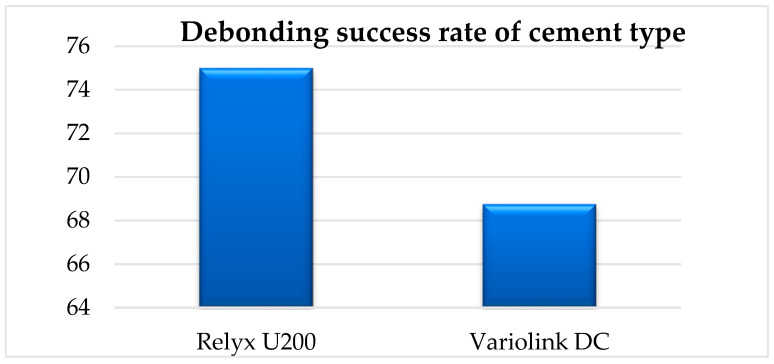
Debonding success rate of cement type.

Referring to other parameters that could influence the effectiveness of the debonding treatment, it can be observed that a large surface area requires a longer laser irradiation time for debonding, but it does not represent a direct factor that influences the outcome of the treatment. According to our results, we can observe that the FPR surface area influences the irradiation time. Another important variable is the type of cement used. In our study, ceramic restorations cemented with RELYX U200 were debonded in 75% of the cases; meanwhile, the same type of ceramic restorations that were cemented with Variolink DC were debonded in a proportion of 69% (Figure 17).

Regarding the material of the prosthetic restorations, the results obtained in our study are the following: ceramic restorations made of layered zirconia were 100% debonded, lithium disilicate ceramic restorations were 87% debonded, feldspathic ceramic restorations were 100% debonded, and ceramic restorations of monolithic zirconia could not be removed using our determined parameters (Figure 15). Pairwise Fisher’s exact tests demonstrated that monolithic zirconia restorations were significantly more resistant to laser debonding than all other materials (*p* < 0.001). In contrast, layered zirconia and feldspathic ceramics exhibited complete success (100%), and lithium disilicate showed a high but slightly reduced success rate (87.5%). Our results are also confirmed by the studies of Morford and Oztoprak [10]. A similar study [27] investigated the debonding efficiency of two types of lasers for bonded lithium disilicate and leucite-based veneers of different thicknesses. Both Er:YAG (600 μm tip, 300 mJ, 6 W, 20 Hz, pulse duration of 100 μs, energy density of 107 J/cm^2^, and 80% water, 60% air) and Er,Cr:YSGG (600 μm tip, at a distance of 2 mm in a non-contact mode for 15 s, 300 mJ, 6 W, 20 Hz, pulse duration of 60 μs, energy density of 107 J/cm^2^, 80% water, 60% air) worked well for glass ceramic debonding, the required time being impacted by ceramic thickness. The debonding time for lithium disilicate was less than that of leucite-based specimens, and the shear bond strength of the retrieved specimens was much lower than that of the control group (no laser group). Overall, Er:YAG worked faster and more efficiently in debonding the prosthetic restorations. Another study [28] compared the ability of Er,Cr:YSGG to debond two types of ceramics, feldspathic and lithium disilicate reinforced glass ceramic veneers, and to measure debonding time and pulpal temperature increase during veneer removal. The authors concluded that lithium disilicate and feldspathic reinforced glass ceramic veneers can be effectively debonded with Er,Cr:YSGG with no uncomfortable temperature increase. The debonding times of these ceramic materials did not differ significantly.

Another comprehensive study [29] was conducted on 200 zirconia specimens, and Er:YAG energies of 80 mJ, 100 mJ, 120 mJ, 140 mJ, 160 mJ, 180 mJ, 200 mJ, 220 mJ, 240 mJ, and 260 mJ (with 10 Hz and 20 Hz) were tested for debonding after cementation with Rely X. Different parameters were observed, such as a change in dentin temperature, surface modifications, and flexural strength. Following laser treatment, neither the dentin nor the zirconia materials showed any surface alterations. The zirconia materials’ flexural strength and surface roughness did not change. The authors concluded that zirconia restorations can be safely and successfully removed using Er:YAG laser treatment, which has a safe temperature change of less than 5.6 °C and does not affect the restorations’ mechanical characteristics. For debonding 3Y-TZP and 5Y-TZP restorations, the ideal frequency and energy settings were determined to be 10/20 Hz and 220 mJ and 10/20 Hz and 200 mJ, respectively. Suliman [30] was also interested in comparing the time required for an Er:YAG laser to remove different types of zirconia and lithium disilicate crowns and reported that the yttria content of the zirconia affected the retrieval time of the crown using the Er:YAG laser; the retrieval time decreased as the yttria content increased. Er:YAG laser debonding of zirconia crowns is a quick, easy, and noninvasive method of crown removal that may be used in clinical settings. A recent study [31] added new light to the problem, comparing recent versus aged bonded ceramic restorations and concluding that the aging approach shortened the removal time for all polymerization procedures. Therefore, at least for the studied lithium disilicate veneers, the laser debonding after a period of intraoral usage is faster than immediate debonding.

The experimental results reported by both our group and other similar studies are also confirmed in clinical trials. El-Damanhoury et al. [32] showed in his clinical study, in which he compared the effects of different Er:YAG laser powers on the pulp temperature and the time required to perform the debonding of lithium disilicate laminate veneers with various thicknesses, that laser power and veneer thickness are crucial; thinner veneers are removed more quickly. The authors concluded that 5.4 W laser power is more effective and results in less pulp temperature variations while debonding thick veneers. Laser applications in dentistry, and in dental prosthetics par excellence, are based on the optical properties of dental materials and the interaction between them and laser radiation. In prosthetics, extensive studies are still needed to establish safe and repeatable clinical protocols. In 2015, Olena Pich and co-authors [33] described the behavior of different dental ceramics (Emax Ceram, Emax ZirCAD and Emax Press) under the action of an Er:YAG laser and a 810 nm diode, concluding that as the thickness of the irradiated sample increases, the laser energy transmitted through the ceramic material drops to 30–40% of its initial levels. Compared to ceramic samples without pigment, those with pigment exhibit a greater loss of laser energy.

These results reinforce the conclusion that ceramic material exerts a strong, categorical influence on the likelihood of successful laser-assisted debonding, with monolithic zirconia being predictably resistant and all other materials demonstrating favorable outcomes.

While Pearson correlation and regression analyses showed no significant association between either thickness (r = −0.067, *p* = 0.76) or surface area (r = −0.045, *p* = 0.84) and debonding time, these results reinforce that geometry alone does not predict procedure duration once debonding is feasible.

Among the limitations of our study, we can mention the relatively small number of samples, the lack of temperature measurement, and the possible reduction in shear bond strength. A newer and important direction of research is represented by the extension of these indications to the level of restorations on implants or implant prosthetic parts.

## 5. Conclusions

This study provides statistically validated evidence that ceramic thickness and ceramic material composition are determinants of Er:YAG laser-assisted debonding. These findings underscore the role of ceramic translucency, crystalline structure, and internal energy absorption in facilitating or impeding laser transmission to the cement interface. Moreover, restoration integrity post-debonding suggests a clinically relevant opportunity for prosthetic reuse. However, the risk of laser-induced fractures underscores the importance of material selection and optimized irradiation protocols to minimize mechanical compromise.

In conclusion, the statistical evidence presented highlights the importance of ceramic thickness as a binary predictor of success, while demonstrating that neither surface area nor thickness linearly affect debonding time. These findings inform restorative planning and support the development of retrievability-aware prosthetic designs and individualized laser protocols in clinical practice.

## Figures and Tables

**Table 1:** Monolithic zirconia thickness measurement.

Sample No.	1						3					
FPR	1			2			1			2		
1/3 C/M/O mm	0.5	1.5	2.2	0.4	0.4	2	0.7	1.3	1.9	0.5	1.4	2.1
Sample no.	6						20					
FPR	1			2			1			2		
1/3 C/M/O mm	0.7	1.1	2.4	0.7	1.2	2.2	0.8	1.3	3.2	0.7	1.6	3

C—cervical third; M—medium third; O—occlusal third.

**Table 2:** Layered zirconia thickness measurement.

Sample No.	7						8					
FPR	1			2			1			2		
1/3 C/M/O mm	0.4	0.8	1	0.6	0.8	2.2	0.4	0.6	1	0.5	0.8	0.8
Sample no.	10						15					
FPR	1			2			1			2		
1/3 C/M/O mm	0.6	0.7	1.1	0.6	0.7	0.9	0.4	0.6	0.8	0.6	0.8	1.1

C—cervical third; M—medium third; O—occlusal third.

**Table 3:** Lithium disilicate thickness measurement.

Sample No.	12						16					
FPR	1			2			1			2		
1/3 C/M/O mm	0.4	0.8	1.1	0.3	0.7	0.7	0.3	0.5	0.8	0.5	0.9	1
Sample no.	17						18					
FPR	1			2			1			2		
1/3 C/M/O mm	0.5	0.9	1	0.5	0.7	0.9	0.8	1.4	1.7	0.4	0.9	1.2

C—cervical third; M—medium third; O—occlusal third.

**Table 4:** Feldspathic ceramic thickness measurement.

Sample No.	2						4					
FPR	1			2			1			2		
1/3 C/M/O mm	0.3	0.6	0.3	0.4	0.6	0.5	0.3	0.7	0.3	0.5	0.6	0.5
Sample no.	11						14					
FPR	1			2			1			2		
1/3 C/M/O mm	0.7	1.0	0.8	0.6	0.7	0.7	0.6	0.8	1.3	0.3	0.7	0.7

C—cervical third; M—medium third; O—occlusal third.

**Table 5:** Sample centralizer.

Sample No.	Restoration No.	Thickness [mm]	Surface [mm²]	Cement Type	Ceramic Type	Debonding	Fissured	Fractured	No Cracks	Debonding Time [s]
1	FPR 1	1.4	95	Relyx	ZM	0				300
	FPR 2	0.9	94	Relyx	ZM	0				300
3	FPR 1	1.3	91	Relyx	ZM	0				300
	FPR 2	1.6	89	Relyx	ZM	0				300
6	FPR 1	1.7	84	Variolink	ZM	0				300
	FPR2	1.7	87	Variolink	ZM	0				300
20	FPR 1	2.1	66	Variolink	ZM	0				300
	FPR 2	2.1	67	Variolink	ZM	0				300
7	FPR 1	0.7	24	Relyx	ZS	1		NO	YES	146
	FPR 2	0.7	25	Relyx	ZS	1		NO	YES	150
8	FPR 1	0.6	42	Relyx	ZS	1		NO	YES	100
	FPR 2	0.7	42	Relyx	ZS	1		NO	YES	102
10	FPR 1	0.8	40	Variolink	ZS	1	Y			37
	FPR 2	0.7	40	Variolink	ZS	1				39
15	FPR 1	0.6	40	Variolink	ZS	1		YES	NO	121
	FPR 2	0.8	40	Variolink	ZS	1		YES	NO	121
12	FPR 1	0.7	54	Relyx	DL	1		NO	YES	17
	FPR 2	0.7	55	Relyx	DL	1		NO	YES	84
16	FPR 1	0.5	54	Relyx	DL	1		YES	NO	150
	FPR 2	0.8	54	Relyx	DL	1		YES	NO	155
17	FPR 1	0.8	54	Variolink	DL	1		NO	YES	85
	FPR 2	0.7	54	Variolink	DL	1		NO	YES	250
18	FPR 1	1	60	Variolink	DL	1		NO	YES	215
	FPR 2	0.8	60	Variolink	DL	0				300
2	FPR 1	0.4	42	Relyx	F	1		YES	NO	121
	FPR 2	0.5	54	Relyx	F	1		YES	NO	121
4	FPR 1	0.4	36	Relyx	F	1		NO	YES	209
	FPR 2	0.5	45	Relyx	F	1		NO	YES	121
11	FPR 1	0.8	36	Variolink	F	1		YES	NO	210
	FPR 2	0.7	45	Variolink	F	1		YES	NO	250
14	FPR 1	0.9	48	Variolink	F	1		NO	YES	32
	FPR 2	0.6	54	Variolink	F	1		YES	NO	110

**Table 6:** Ceramic type restoration debonded or not debonded.

Ceramic Type	Debonded	Not Debonded	Total
Monolithic Zirconia	0	8	8
Layered Zirconia	8	0	8
Lithium Disilicate	7	1	8
Feldspathic Ceramic	8	0	8
Total	23	9	32

**Table 7:** Fisher’s exact test results between ceramic types.

Comparison	Debonding Rate (%)	*p*-Value	Statistically Significant	Comments
MZ vs. LZ	0% vs. 100%	<0.001	Yes	Layered zirconia is highly responsive to laser; monolithic zirconia is completely resistant
MZ vs. F	0% vs. 100%	<0.001	Yes	Feldspathic ceramics are fully retrievable; monolithic zirconia is not
MZ vs. LD	0% vs. 87.5%	<0.001	Yes	LD shows high retrievability, still significantly better than MZ
LZ vs. LD	100% vs. 87.5%	≈1.0	No	Both materials show high success; difference not statistically meaningful
LZ vs. F	100% vs. 100%	1.0	No	Identical outcomes: both are excellent candidates for laser debonding
LD vs. F	87.5% vs. 100%	1.0	No	LD has one failed case, but the difference from F is not statistically significant

## Data Availability

The data presented in this study are available on request from the corresponding author.

## Abbreviations

The following abbreviations are used in this manuscript:

FPR	Fixed prosthetic restorations
Er:YAG	Erbium-doped yttrium-aluminum-garnet
Er,Cr:YSGG	Erbium-chromium yttrium-scandium-gallium-garnet
MZ	Monolithic zirconia
LZ	Layered zirconia
LD	Lithium disilicate
F	Feldspathic

## References

[1] Girard J.L. (2014). Advancement in the Removal of Permanently Cemented Crowns and Bridges. Guident.

[2] Al Moaleem M.M. (2016). Systems and techniques for removal of failed fixed partial dentures: A review. Am. J. Health Res..

[3] Bajunaid S.O. (2017). Review of techniques for the intact removal of a permanently cemented restoration. Gen. Dent..

[4] Sharma A., Rahul G.R., Poduval S.T., Shetty K. (2012). Removal of failed crown and bridge. J. Clin. Exp. Dent..

[5] Liebenberg W.H. (1995). Methods for removing crowns and bridges: Preserving the restoration. Quintessence Int..

[6] Walid A.J. (2020). Application of Laser Technology in Fixed Prosthodontics—A Review of the Literature. Open J. Stomatol..

[7] Gupta A. (2015). A Simple Chairside Technique of Removing Crown and Fixed Partial Denture Restorations. J. Dent. Mater. Tech..

[8] Deeb J.G., Grzech-Leśniak K., Brody E.R., Matys J., Bencharit S. (2022). Erbium laser-assisted ceramic debonding: A scoping review. J. Prosthodont..

[9] Bernal C., Ryoung Lee E.M., Eduardo C.D.P., Souza A.M.A., Azevedo L.H. (2021). Retreatment of 6 Ceramic Restorations in a Single Session—The Application of Er:YAG Laser And CAD/CAM Technology: An 1 Year Follow Up Clinical Evaluation. Braz. Dent. Sci..

[10] Morford C.K., Buu N.C., Rechmann B.M., Finzen F.C., Sharma A.B., Rechmann P. (2011). Er: YAG laser debonding of porcelain veneers. Lasers Surg. Med..

[11] Rechmann P., Buu N.C., Rechmann B.M., Le C.Q., Finzen F.C., Featherstone J.D. (2014). Laser all-ceramic crown removal—A laboratory proof-of-principle study—Phase 1 material characteristics. Lasers Surg. Med..

[12] van As G.A. (2013). Using the Erbium Laser to Remove Porcelain Veneers in 60 Seconds Minimally Invasive. Efficient, and Safe. J. Cosm. Dent..

[13] Spath A., Smith C. (2017). Removal of Modern Ceramics. Compendium.

[14] Gozneli R., Sendurur T. (2023). Er:YAG laser lithium disilicate crown removal: Removal time and pulpal temperature change. Lasers Med. Sci..

[15] Kellesarian S.V., Ros Malignaggi V., Aldosary K.M., Javed F. (2018). Laser-assisted removal of all ceramic fixed dental prostheses: A comprehensive review. J. Esthet. Restor. Dent..

[16] Zhang X., Dong H., Wu X., Li Q., Zhao J. (2024). Effects of Er:YAG laser debonding on changes in the properties of dental zirconia. PLoS ONE.

[17] Ghazanfari R., Azimi N., Nokhbatolfoghahaei H., Alikhasi M. (2019). Laser Aided Ceramic Restoration Removal: A Comprehensive Review. J. Lasers Med. Sci..

[18] Jeha B.A., Haddad R. (2025). What is the most effective method for reducing pain during debonding procedures? A systematic review. Int. Orthod..

[19] Karagoz-Yildirak M., Gozneli R. (2020). Evaluation of rebonding strengths of leucite and lithium disilicate veneers debonded with an Er:YAG laser. Lasers Med. Sci..

[20] El-Sheikh N.A., Wahsh M.M., Hussein G.A. (2024). Laser debonding of ultrathin occlusal veneers fabricated from different CAD/CAM ceramic materials. BMC Oral. Health.

[21] Zhang X., Dong H., Guo C., Zhang X., Zhang D., Wu X., Zhao J. (2021). Effects of laser debonding treatment on the optical and mechanical properties of all-ceramic restorations. Lasers Med. Sci..

[22] Abo Zaid A., Ebeid K., Wahsh M., El Demellawy M. Effect of Er,Cr: YSGG laser debonding treatment on the optical properties and surface roughness of ceramic laminate veneers: An in vitro study. J. Prosthodont..

[23] Deeb J.G., Bencharit S., Dalal N., Abdulmajeed A., Grzech-Leśniak K. (2019). Using Er:YAG laser to remove lithium disilicate crowns from zirconia implant abutments: An in vitro study. PLoS ONE.

[24] Elkharashi A., Grzech-Leśniak K., Deeb J.G., Abdulmajeed A.A., Bencharit S. (2020). Exploring the use of pulsed erbium lasers to retrieve a zirconia crown from a zirconia implant abutment. PLoS ONE.

[25] Birand C., Kurtulmus-Yilmaz S. (2022). Evaluation of Er,Cr:YSGG laser irradiation for debonding of zirconia hybrid abutment crowns from titanium bases. Lasers Med. Sci..

[26] Deeb J.G., Grzech-Lesniak K., Bencharit S. (2023). Evaluation of the effectiveness and practicality of erbium lasers for ceramic restoration removal: A retrospective clinical analysis. PLoS ONE.

[27] Eid H., Zohdy M.M., Ghali R.M., Salah T. (2021). Efficiency of Er:YAG laser versus Er;Cr:YSGG laser in debonding of different glass ceramic veneers: An in vitro study. Laser Dent. Sci..

[28] Alikhasi M., Monzavi A., Ebrahimi H., Pirmoradian-najafabadi M., Shamshiri A., Ghazanfari Hashemi R.S. (2019). Debonding Time and Dental Pulp Temperature with the Er,Cr:YSGG Laser for Debonding Feldespathic and Lithium Disilicate Veneers. J. Laser Med. Sci..

[29] Jiang L., Li X.Y., Lu Z.C., Yang S., Chen R., Yu H. (2024). Er:YAG laser settings for debonding zirconia restorations: An in vitro study. J. Mech. Behav. Biomed. Mater..

[30] Suliman S., Sulaiman T.A., Deeb J.G., Abdulmajeed A., Abdulmajeed A., Närhi T. (2024). Er:YAG laser debonding of zirconia and lithium disilicate restorations. J. Prosthet. Dent..

[31] Yıldız P., Güneş Ünlü D., Talay Çevlik E., Üşümez A. (2022). Removal of lithium disilicate veneers with Er,Cr:YSGGL laser: Now? Or after ageing?. Lasers Med. Sci..

[32] El-Damanhoury H.M., Salman B., Kheder W., Benzina D. (2022). Er:YAG Laser Debonding of Lithium Disilicate Laminate Veneers: Effect of Laser Power Settings and Veneer Thickness on The Debonding Time and Pulpal Temperature. J. Lasers Med. Sci..

[33] Pich O., Franzen R., Gutknecht N., Wolfart S. (2015). Laser treatment of dental ceramic/cement layers: Transmitted energy, temperature effects and surface characterisation. Lasers Med. Sci..

